# Vaccination

**Published:** 1821-03-01

**Authors:** 


I. *A Statement of Facts, tending to establish an Estimate of the true value and present state of Vaccination.* By Sir GILBERT BLANE, Bart. &c. &c. London. T. and G. Underwood.

II. *Copy of the Report to the Secretary of State for the Home Department, from the National Vaccine Establishment; dated the* 18*th of May*, 1820.

III. *Circular, addressed to Practitioners in general, respecting Varieties and Modifications of the Vaccine Pustules, occasioned by Herpetic and other Eruptive states of the Skin.* By E. JENNER, M.D. &c. &c. 1821.

WHEN two individuals commence opposite trains of reasoning on subjects of medical science, it is not very rare for both to find that they have proceeded by that erroneous process which, after a great expenditure of words, terminates in their arriving at the post from which they started,—and discovering that the only mode which can bring the question to any issue at all, is precisely such as will admit of no manner of contention. Facts, says Lord Bacon, are the prelusions to stability. Sir G. Blane's paper is a specification of these, with as little speculation, or imagination, as any thing we have seen. It was first published in the Medico-Chirurgical Transactions, but as it would there have gravely reposed "above the limits of a vulgar fate," it has been wisely translated into a more vendible and locomotive form, and we trust, for the sake of truth, that it will make an extensive excursion throughout the inquisitive ranks of society in all parts of the globe.

The object of this excellent paper, is to prove the sensible effects of vaccination in diminishing the mortality from small-pox, and previous to advancing any proofs of which, Sir Gilbert very candidly appretiates the claims of variolus inoculation:—

> "But it has no tendency, like vaccination, to extirpate the disease, and from the impossibility of rendering it universal, it has actually been found to add to the general mortality of small-pox, by opening a new source for the diffusion of its virus."*

To effect his main object, a comparison of the mortality of small-pox, as influenced by vaccination as well as by ino-

---

* We may not inaptly ask those who may think highly of the innocence of small pox inoculation, whether the great fire of London might not have been caused by kindling a few shavings?*

* See Report.

culation from itself, he selects from the bills of mortality four periods, each of fifteen years, for the purpose of exhibiting the mortality of small-pox in each of these series in regard to each other. The result of these computations stands as follows.

"Ratio of the mortality of small-pox to the total mortality.

From 1706 to 1720; one in 12.7 ; that is 78 in 1000
—— 1745 to 1759; one in 11.2; that is 89 in 1000
—— 1785 to 1798; one in 10.6 ; that is 94 in 1000
—— 1804 to 1818; one in 18.9 ; that is 53 in 1000" P. 5.

*Fractions are not noticed in the last column of numbers.*

There is one fallacious inference which might naturally follow the consideration of this estmimate, viz.

"That the proportion of deaths from small-pox to the total mortality increased in the course of the last century; so that inoculation appears to have added to the mortality."

This, however, is not exactly fair, for "in the course of that century the general mortality itself was greatly diminished in relation to the population."

The diminution is attributed to the preservation of infant life under the age of two years;—

"Owing chiefly to the improvements in ventilation and cleanliness, but greatly also to laying aside the custom of exposing them to the open air in winter and early in spring; either from inadvertency, or from the false notion of rendering them hardy, whereas they catch inflammation of the lungs. Nothing tends more to the health, strength, and growth of children, than genial warmth. It seems chiefly owing to the great plenty and cheapness of fuel, that the race of people in Lancashire are so superior in their form and size. In Buckinghamshire, on the contrary, where the fuel is extremely scanty and dear, the race of people is small and puny, insomuch that it is provided by Act of Parliament that men shall be admitted into the militia of smaller stature in this than other counties."

Sir G. consequently considers that the relative mortality of small-pox to the total mortality should be viewed in its proportion to the increase or decrease of population. As the population of the metropolis nearly doubled in the last century it might be thought that it had decreased, but this increase having ensued in the parishes of Mary-le-bone and St. Pancras, *without* the bills, and the increase in the west, being caused by migration from the east, &c. the offset is such that,—"the addition to the population, if any, *within* the bills of mortality, does not seem to be so considerable as to affect the computation."

Thence it would appear, that inoculation did not add so much to metropolitan mortality; but it is also clear that it had that effect in situations where pestilence was introduced by the zeal for inoculation.—Assuming that the given proportions of variolus deaths had proceeded unchecked by vaccination, it results from these calculations, that the very reverse of the following proposition must have been afforded.

" Even under the very imperfect practice of vaccination which has taken place in this metropolis, 23134 lives have been saved in the last fifteen years, according to the best computation that the data afford." 7.

A fluctuating mortality from small-pox continues open to every grade in the scale, from loose habits of perpetuating inoculation in the small-pox hospitals, as the increase of deaths in 1805 exhibits.* Sir G. Blane then accumulates abundant "irrefragable historical evidence" to prove the competence of vaccination to total extirpation of small-pox, a point which he labours with as much force of reasoning as felicity of language.

Small-pox, by recurrence, affords an occasional violation of a general rule; the exceptions after vaccination are probably more frequent still, and instead of forming an objection, must be considered by great minds as an urgent reason for universality of vaccination, which would enclose those within the boundaries of protection, whose susceptibilities are peculiar, if such susceptibilities have actual existence. Small-pox, in continuation of this argument, destroys a hundred "for every one that perishes by the plague;" it is therefore the "greatest known scourge." Some of the soundest political economists alledge—

" That small-pox does not diminish the numbers of mankind, nor vaccination increase them; for population is determined by subsistence, and the indefinite powers of procreation soon repair the ravages of disease. But however true this may be, the miseries incident to so many of those who survive small-pox, whereby they become a burden to themselves, their families, and to society, render this disease uncontrovertibly an evil of the first magnitude,—not to mention the intense sufferings and afflictions inseparable from it."

Our author justly laments the inefficacy of legislative regulations to restrain inoculation, and indiscriminate exposure of inoculated individuals in the general community. We have passed our comments on this, in the review of Mr. Cross's valuable book, and we cannot but lament the kind of spirit and intelligence, which has been of late displayed by a

---

* " Seven hundred and twelve persons are reported by the bills of mortality of London, to have died of Small-pox within the last year." —*Rep.* p. 3.—*Sir G. Blane's Statement*, p. 14.

magistrate at a public office, when applied to upon an illegal transaction of this nature.*

After the demonstration of the possibility of extirpation, it remains to be asked whether alledged failures operate to render the practice inexpedient.† This resolves itself into an inquiry into the history of these failures.

"The fifth day is the most common limit of this disorder, it sometimes stop short on the third; sometimes not till the sixth or seventh; and in a very few cases it has been known to run the common course of small-pox. What forms the strong line of distinction from proper small-pox, is that, with a few exceptions, it does not advance to maturation and secondary fever, which is the only period of danger. I am not prepared to deny that death may have occurred in a few instances; nay, there seems sufficient evidence that it actually has; but these adverse cases are so rare, as not to form the shadow of an objection to the expediency of the general practice." P. 11.

The replies made to Sir Gilbert at a meeting of those "extensive and eminent practitioners" who form the Medico-Chirurgical Society, furnishing his enquiries with *two* fatal cases only of after small-pox, are very decisive and satisfactory, and render it more extraordinary that the exception should ever become the rule with men of judgment.

We shall not pause long on the arguments for universal vaccination; for however we may be fascinated for a moment with the helpful ornament which the writer hath lent to the conclusion of this important question, we see but a thousandth repetition of that half-coaxing and half-boring persuasion which avails so little with our countrymen. We would fain hope that the attainment of the *maximum*, that is, total extirpation, should not be impracticable and hopeless, and that the unceasing havoc of small-pox would yet cease to affect the destinies of the world; and we feel that the utility of vaccination in the army and navy has shewn us what may be reasonably expected from such a vigorous effort in private practice.

"Their example has been by no means followed among the civil population of England." No! nor ever will, unless one hundred years of slavery, which we would be the last to propose, should bring over some modification of the national character. But the most enlightened, wise, and reflecting people in the world, are very suspicious of all schemes for their benefit, and never approve of gratuitous advantages. John Bull hates to be taught, and thinks obstinacy his great-

---

* Bow Street Report, Morning Chronicle, Dec. 28, 1820.

† Report, p. 3.

est virtue. We will indulge, however, in a single *argumentum ad hominem*, and give him the irreverend council of his sagacious, though never very cordial, friend from the other side of Tweed—"get rid of the De'el, man, and there will soon be no Lord." This repartee, which we quote more for its wit than piety, may be precisely applied to vaccination; and if those who are inimical to its interests, would only unite with its friends in making its diffusion complete, we are sensible that the frequent demands for it would soon cease to grieve the eye of the former.

A late emanation from the masters and court of assistants of the Royal College of Surgeons, is a measure of which we cannot but approve, renewing their engagements to persevere in exclusive vaccination, and recommending the same conduct to their ordinary members. The Board of the National Vaccine Establishment has undertaken to circulate these through the medium of their various members throughout the united kingdom; and, considering the severe test which vaccination has surmounted, why should there not be corresponding resolutions formed by all the county-hospitals, dispensaries, and other provincial institutions, tending to recommend their example to their neighbours, to extend the practice of vaccination, to win back the disingenuous, and support the weak and irresolute. An association of this nature, called the Gloucestershire Vaccine Association, for promoting cow-pox and discouraging small-pox inoculation, was formed at Gloucester, in April 1810, consisting of the sixty-three mose eminent medical men in the county. At the head of this body stood a man of powerful faculties and comprehensive views, Dr. Baron, who, while others gazed supinely or submitted ingloriously to fatality, formed a circumvallation around him, which, if it had continued well defended, would never have been passed again by the invading enemy.* Such measures might restrain inoculation, more effectually than acts of legislation; for there are many instances in which a man is more deterred from doing wrong by his pride than moral sense.

The report of the Board for 1819, published May 1820, states, that "the practice of vaccination in His Majesty's dominions continues to advance, notwithstanding many unfavourable occurrences." Gloucester, which is stated to have

* See Edinburgh Medical Journal (about this period) containing the resolutions, recommending vaccination exclusively, except in cases of extreme necessity; for example, if the small-pox breaks out among persons who have never had that disease, where no vaccine matter can be obtained.

been free from small-pox since the year 1817 by the report, has lately however exhibited some disgraceful spectacles.

"The boroughs of Clomnell and Newton Limavady, in Ireland, and Mothvey in Caermarthenshire, with the whole country for twenty miles around it, are reported to have completely succeeded in the extirpation of the small-pox; and in the island of Guernsey only one solitary case of that fatal distemper is known to have occurred during the last year."

Exotic vaccination thrives prodigiously, legal enactments having extinguished small-pox in Denmark for the last eight years. These enactments are extended to the Danish West India colonies. Austria has nearly extirpated it, and the circle of Rezat in Bavaria, containing half a million of people, *entirely* since 1807. Some proofs are given of the *life-long* security* of vaccination. The report also shews that 30,000 *vaccinés* in five different counties have furnished *no* exception. The register of the Small Pox Hospital of London furnishes one exception out of 46,662 cases; the Foundling Hospital out of 19 years vaccination, two mild cases; Royal Military Asylum none; the National Vaccine Establishment, out of 60,000, reports five. Having represented the favourable side of the profile, they point out the event of great numbers of vaccinated persons having had varioloid affections, twenty-one cases of secondary small-pox, and many of small-pox after neither, proving very fatal, "attributable to the neglect of universal vaccination," and the "too frequent practice of small-pox inoculation." The failures of vaccination and variolation are in part attributed to "certain peculiarities of constitution, that will exempt some individuals from all common laws." One word to the wise, as to these rare susceptibilities and failures, without entering into the grand question, in what manner varioloids are made, for which there is neither time nor place at present. Let us examine, for a moment, the ordinary mode of reporting these *infortunia;*—vaccination was done by such a person, perhaps a celebrated surgeon, this is a common answer after a failure,

* Sir G. Blane seems inclined to admit the omnipotency of time in weakening the virtue of vaccination, as well as of other things. The laws of the human constitution are full of diversity, but we are as yet reluctant to admit that which is as much at variance with the analogies of small-pox inoculation, as with much direct evidence. Perhaps these exceptions, from which a legal personage was pleased to term vaccination a lease for a term of years only, may very probably arise out of some of the minute impediments which attend the process of vaccination. We believe we have heard that the discoverer has been able to detect the cause.

and general celebrity is, of course, a satisfactory proof of minute and particular knowledge. "Peculiarities of constitution" is another *say-something*, but this must be owing to a cause; cutaneous diseases were pointed out as impediments, by Dr. Jenner, as early as 1804; but, by some means or other, his precautions have been but little attended to, so that we find them repeated in 1821. Dr. Jenner in the London Medical and Physical Journal of August 1804, in Dr. Willan's Essay on Vaccination, and in an appendix to Dr. Wilson Philip's work on Febrile Diseases, stated that certain cuticular affections have the property of creating such deviations as limit or wholly counteract security. If such deviations are formed, where does the principle terminate? not within a very narrow compass. We have seen these facts repeatedly illustrated, and feel almost shocked at that neglect of minutiæ in this respect, which is so unpardonable in men of science, and attention to which generally constitutes the perfection of skill in our profession. Mr. Cross gives five cases of small-pox after vaccination in his work, out of which three establish Dr. Jenner's positions.* We here briefly analyze the whole 1*st Case.* Regular distinct small-pox after vaccination—vaccinated three or four years before "by an *eminent* surgeon, who *believed* the cow-pox to be satisfactory." "Two vaccinated children from the same family were inoculated from him without effect." 2d Case, similar.—"*Five* others of the same family had been vaccinated, and *were unaffected by the contagion.*" 3d Case—"The mother states that *several pimples* came out upon the right arm at the time of vaccination, one of which has left a slight scar. There is a *large* scar from vaccination." "Her brother, vaccinated ten years, was inoculated from her *without inconvenience*," 4th Case. Fatal patechial small pox. Preceding circumstances precisely similar to the last case. 5th Case, Fatal confluent small pox. One vaccine vesicle only had been formed, from which *ichor was* taken to vaccinate others. In the first two cases there is no external evidence of irregularity, but the protection afforded at the same time to other individuals of the same family, prompts us to doubt whether the cause of failure might not have been obviated—and why should super-vaccination ever be neglected? In the 3d and 4th cases eruptions are stated to have appeared, and scarred simultaneously with vaccination; the original vaccine vesicle leaving a *broad* scar.—These eruptions which pre-existed in the constitution, be-

---

* Pp. 58 to 65.

came coincident in development with the vaccine vesicle, and it generally happens that if they are not subdued before or during the vaccine process, the vaccine vesicle may be so far rendered incorrect, by a reciprocal process, which alters the secreting action of both, that imperfect security results. The large "*scars*," in these two cases of failure, instead of denoting increased security, as ignorance imagines, are truly emblematic of the rambling deviation of the vaccine pustule, under the influence of eruptive diseases, unless in those cases where a very broad shining superficial mark is produced by rubbing off the scab in the last stages, which is generally level with the cuticle, and is evidently cuticular only. How can we be surprized at small-pox after vaccination, when it was the custom for years to confide in one vesicle, and to alter its course by robbing that to inoculate others ?—*V. Case* 5.

We hope that this short article will be found to contain some useful practical intelligence, and much compressed analysis. At all events, we regard it favourable to the interests of vaccination, that the clamorous effusions of party are changing into the cool discussions of men of science.

